# Senolytic activity of small molecular polyphenols from olive restores chondrocyte redifferentiation and promotes a pro-regenerative environment in osteoarthritis

**DOI:** 10.18632/aging.103801

**Published:** 2020-08-03

**Authors:** Marta Varela-Eirín, Paula Carpintero-Fernández, Agustín Sánchez-Temprano, Adrián Varela-Vázquez, Carlos Luis Paíno, Antonio Casado-Díaz, Alfonso Calañas Continente, Virginia Mato, Eduardo Fonseca, Mustapha Kandouz, Alfonso Blanco, José Ramón Caeiro, María D. Mayán

**Affiliations:** 1 CellCOM Research Group, Instituto de Investigación Biomédica de A Coruña (INIBIC), Servizo Galego de Saúde (SERGAS), Universidade da Coruña (UDC), Xubias de Arriba, A Coruña, Spain; 2 Neurobiology-Research Service, Hospital Universitario Ramón y Cajal (IRYCIS), Madrid, Spain; 3 UGC Endocrinology and Nutrition, Maimónides Biomedical Research Institute of Córdoba (IMIBIC), Hospital Universitario Reina Sofía – CIBERFES, Universidad de Córdoba, Córdoba, Spain; 4 Centre for Medical Informatics and Radiological Diagnosis, Universidade da Coruña, A Coruña, Spain; 5 Department of Pathology, School of Medicine, Wayne State University, Detroit, MI 48202, USA; 6 Flow Cytometry Core Technologies, UCD Conway Institute, University College Dublin, Dublin, Ireland; 7 Department of Orthopaedic Surgery and Traumatology, Complexo Hospitalario Universitario de Santiago de Compostela (CHUS), Universidade de Santiago de Compostela (USC), Choupana s/n, Santiago de Compostela, Spain

**Keywords:** senescence, dedifferentiation, osteoarthritis, connexin43, tissue regeneration

## Abstract

Articular cartilage and synovial tissue from patients with osteoarthritis (OA) show an overactivity of connexin43 (Cx43) and accumulation of senescent cells associated with disrupted tissue regeneration and disease progression. The aim of this study was to determine the effect of oleuropein on Cx43 and cellular senescence for tissue engineering and regenerative medicine strategies for OA treatment. Oleuropein regulates Cx43 promoter activity and enhances the propensity of hMSCs to differentiate into chondrocytes and bone cells, reducing adipogenesis. This small molecule reduce Cx43 levels and decrease Twist-1 activity in osteoarthritic chondrocytes (OACs), leading to redifferentiation, restoring the synthesis of cartilage ECM components (Col2A1 and proteoglycans), and reducing the inflammatory and catabolic factors mediated by NF-kB (IL-1ß, IL-6, COX-2 and MMP-3), in addition to lowering cellular senescence in OACs, synovial and bone cells. Our *in vitro* results demonstrate the use of olive-derived polyphenols, such as oleuropein, as potentially effective therapeutic agents to improve chondrogenesis of hMSCs, to induce chondrocyte re-differentiation in OACs and clearing out senescent cells in joint tissues in order to prevent or stop the progression of the disease.

## INTRODUCTION

Articular cartilage from patients with osteoarthritis (OA) shows accumulation of dedifferentiated and senescent cells [1–4] together with increased inflammation and breakdown of cartilage extracellular matrix (ECM) [5]. Importantly, cartilage and synovial tissue from OA patients contain high levels of the gap junction protein connexin43 (Cx43). Targeting Cx43 might be a promising approach to treat several age-related and chronic degenerative diseases by modulating tissue regeneration, inflammation and response to injury [6]. Cx43 belongs to the integral membrane protein family called connexins, that enable direct communication between neighboring cells via hemichannels, gap junctions, extracellular vesicles and tunneling nanotubes [7]. Additionally, connexins act as signaling hubs regulating different signaling pathways via their cytoplasmic domains [2, 8]. Cx43 is the major Cx protein expressed in chondrocytes, synovial cells (SC) and bone cells (BC) [9–11] and it has been involved in normal development and function of joint tissues [12, 13] and joint disorders [9–11, 14, 15]

During tissue regeneration and following injury, dedifferentiation, redifferentiation and senescence processes play finely tuned temporal and spatial roles to reverse the loss [2, 16]. In addition, accumulation of senescent cells is described to play a major role in OA progression [1, 17–22]. Cellular senescence is a stable cell-cycle arrest with increased expression of cell cycle inhibitors such as p16^INK4A^ and enhanced synthesis of the senescence-associated secretory phenotype (SASP) factors, which mainly consist of inflammatory cytokines and ECM degrading enzymes, among other factors [23]. The elimination of senescent cells using senolytics has been described to attenuate cartilage and joint degeneration in OA [1, 24–26]. In fact, some of these drugs including UBX0101 and the natural-occurring flavone Fisetin [27, 28] with potential senolytic activity are currently under clinical trial for OA treatment (NCT04210986; NCT04349956; NCT04229225; NCT04129944) [29–32].

Cx43 has been involved in different phases of tissue regeneration including chronic inflammation, cell differentiation and cellular senescence [33, 34]. Overexpression of Cx43 and enhanced gap junction intercellular communication (GJIC) in osteoarthritic chondrocytes (OACs) compromise their ability to re-differentiate, promoting a stem-like state by activating Twist-1, which leads to dedifferentiation via epithelial-to-mesenchymal transition (EMT) [2]. However, we have also previously observed that high levels of Cx43 lead to p53/p21-mediated cellular senescence [2]. Increased levels of Cx43 in OA cartilage is extensively described in the literature [9, 11, 35–37], as well as the presence of dedifferentiation (stem-like state) and proliferative chondrocytes [4, 38–44] together with the accumulation of senescent cells [1, 18, 20, 29], indicating that both phenotypes, dedifferentiated and senescent chondrocytes, co-exist in cartilage from OA patients and contribute to the progression of the disease. However, we have recently demonstrated that downregulation of Cx43 reduces stemness and accumulation of senescent cells, indicating that Cx43 acts as a molecular switch in the phenotype of chondrocyte within a wound healing process [2]. In fact, downregulation of Cx43 in different wound healing and age-related disorders halts disease progression by restoring tissue regeneration [45–47].

Oleuropein is the most abundant polyphenol in the leaves and fruit of the olive plant and is a potent antioxidant agent with anti-tumour and anti-inflammatory properties [48, 49]. The mechanism of action of this polyphenol is under investigation [48]. Some studies showed that oleuropein and its major metabolite hydroxytyrosol have antioxidant activity by inhibition and/or scavenging of ROS [50], which can reduce NF-kB activation [51, 52]. Other mechanistic studies implicate nitric oxide (NO) production [53, 54] or autophagy and inhibition of the mammalian target of rapamycin (mTOR) [55, 56]. A gene expression profiling study has suggested that oleuropein affects the expression of genes involved in oxidative stress, inflammation, fibrosis, cell proliferation or differentiation [57], suggesting that the beneficial effects of this molecule may be multifactorial and context-dependent [57]. Innovative approaches based on functional foods have been recently studied and many beneficial effects of dietary olive oil on human health were already described [58–62]. In fact, extra virgin olive oil and olive leaf extract supplemented diets have been shown to reduce inflammation and preserve cartilage, muscle and joint function in rat preclinical models of OA [63–68].

In this study, we have used a small-scale screening to identify compounds that downregulate Cx43. Here we describe the use of small molecules based on olive phenolic compounds to downregulate Cx43 in OA by using 2D and 3D human cartilage models. We have found that oleuropein decreases Cx43 promoter activity and GJIC, thus enhancing osteogenesis and chondrogenesis in hMSCs and redifferentiation of OACs. Beside downregulation of Cx43, olive-derived small polyphenols reduce cellular senescence in OACs, BC and SC, in addition to inflammatory and catabolic activities related to cartilage degradation in OA patients.

## RESULTS

### Olive-derived polyphenols impair adipogenesis and enhance the chondrogenic and osteogenic ability of hMSCs

**Figure 1:**
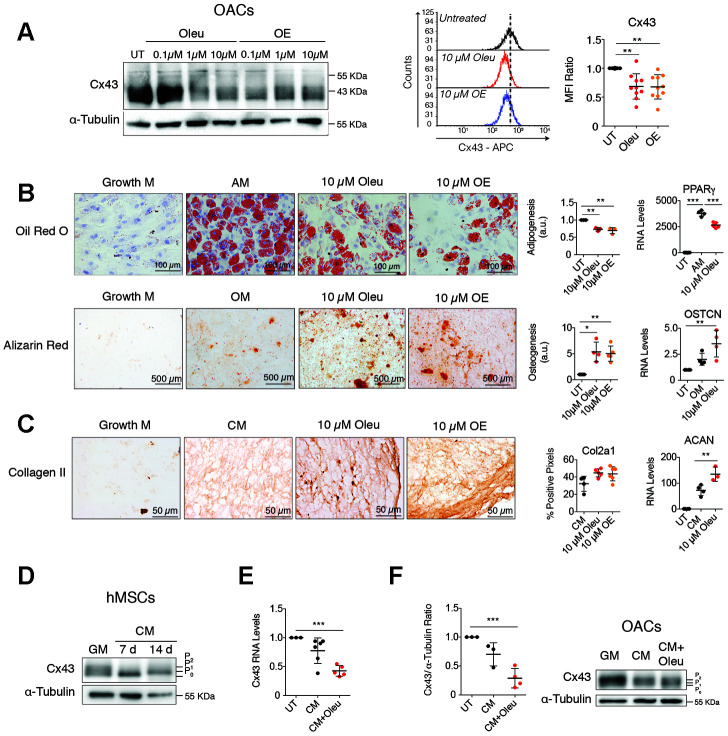
**Downregulation of Cx43 during chondrogenesis improves differentiation towards chondrocytes.** (**A**) Treatment of OACs with oleuropein (Oleu) or olive extract (OE) for 2 h significantly downregulates Cx43 protein detected by western-blot and flow cytometry. Median fluorescence intensity (MFI) ratios of oleuropein and OE treatments with respect to their untreated controls of each experiment are represented (n=10 independent experiments, *P*=0.0003). (**B**) Differentiation capacity of hMSCs isolated from bone marrow grown in adipogenic (top, 21 days) or osteogenic (bottom, 21 days) medium supplemented with 10 μM oleuropein or 10 μM OE. hMSCs cultured in growth medium were used as a control. Top, adipogenic evaluation by oil red O for lipid staining and by PPARγ gene expression. Data represent the ratio of cells containing lipid deposits to the total number of cells (n=3 independent experiments, *P*<0.0001). Values were normalized to hMSCs differentiated in adipogenic medium without treatment (AM). On the right, PPARγ gene expression (n=4 independent experiments, *P<*0.0001). Alizarin red staining was used to detect calcium deposits for osteogenic differentiation. Values were obtained by counting red pixels and normalized to those of hMSCs differentiated in osteogenic medium without treatment (OM) (n=4-6 independent experiments, *P*=0.0317). OSTCN gene expression was measured to confirm osteogenic differentiation (n=4 independent experiments, *P*=0.0055). (**C**) Differentiation capacity of hMSCs isolated from bone marrow grown in chondrogenic medium as micromasses for 30 days. Representative images for Col2A1. The quantification is shown on the right (n=5–6 micromasses from independent experiments, *P*=0.0423). Chondrogenesis was also evaluated by ACAN gene expression quantification (n=3–4 independent experiments, *P*<0.0001). (**D**) Cx43 protein levels in hMSCs, isolated from bone marrow and from inguinal fat, differentiated for 7 and 14 days in the presence of chondrogenic medium (CM) in comparison to untreated hMSCs cultured in normal growth medium (GM). (**E**) Cx43 RNA expression of hMSCs cultured for 14 days in the presence of chondrogenic medium (CM) alone or supplemented with 10 μM oleuropein. Data were normalized to HPRT-1 levels (n=5-6 independent experiments, *P*<0.0001). (**F**) Cx43 protein levels were analyzed by western blot in OACs differentiated for 7 days in the presence of chondrogenic medium (CM), supplemented with 10 μM oleuropein. The graph represents the quantification from 3 independent experiments (*P*=0.0004). Data is expressed as mean±SD, one-way ANOVA; *P<0.05, **P<0.01 and ***P<0.0001.

We used a small-scale screening to identify compounds that downregulate Cx43 (data not shown), and we identified the small molecule oleuropein (Figure 1A). Oleuropein and an olive-extract (OE) significantly reduced Cx43 protein levels in OACs (Figure 1A). MTT assays showed no effect of 0.1-10 μM oleuropein on cell viability of primary chondrocytes and hMSCs (Supplementary Figure 1). Based on these results and other studies [69], 10 μM was selected as the highest concentration with no toxic effect after 17 h in culture.

We next examined hMSCs differentiation capacity in the presence of oleuropein (Figure 1B, 1C and Supplementary Figure 2). In accordance with previous results [69], oleuropein-treated hMSCs showed significantly less adipogenic differentiation ability, whereas osteogenesis was significantly increased (Figure 1B and Supplementary Figure 2A, 2B). Furthermore, a 3D micromass culture system using chondrogenic medium supplemented with 10 μM oleuropein or OE revealed increased ECM properties, reflecting a greater degree of chondrogenic differentiation, with higher levels of Col2A1 deposition and increased levels of aggrecan (ACAN) gene expression (Figure 1C and Supplementary Figure 2C). Remarkably, decreased Cx43 gene expression was detected in hMSCs during differentiation, mainly during chondrogenesis (Figure 1D, 1E and Supplementary Figure 3A). We have also observed changes in Cx43 phosphorylation pattern at 7 and 14 days of chondrogenic differentiation (Figure 1D), which can affect Cx43 stability and channel activity. Different Cx43 phosphorylation patterns were also detected during adipogenesis and osteogenesis, suggesting differential regulation of Cx43 during hMSCs differentiation (Supplementary Figure 3B). The treatment of hMSCs during chondrogenic differentiation with oleuropein for 14 days caused an additional 1.7 fold reduction in Cx43 gene expression (Figure 1E).

**Figure 2:**
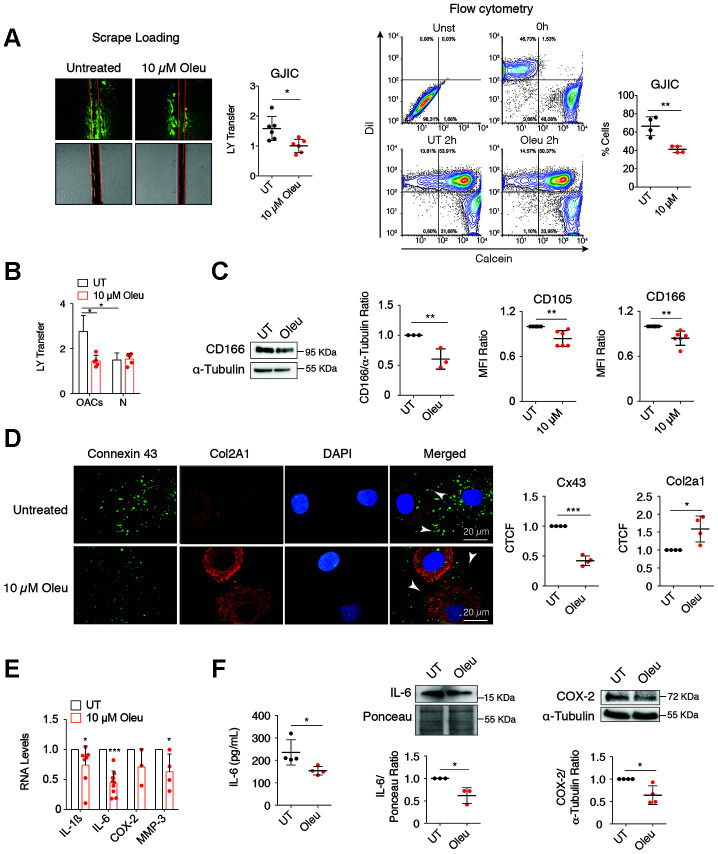
**Downregulation of Cx43 by oleuropein decreases GJIC and improves the phenotype of OACs.** (**A**) Oleuropein (Oleu) treatment significantly decreases GJIC evaluated by an SL/DT assay when OACs were exposed with this molecule for 2 h (top, n=6 independent experiments; Student’s *t* test, *P*<0.0001). The results were confirmed by calcein transfer by flow cytometry (n=4 independent experiments; Student’s *t* test, *P*=0.0037). (**B**) Graph showing the effect of oleuropein on GJIC when healthy chondrocytes (N) were exposed to 10 μM oleuropein compared with OACs (n=5 independent experiments; one-way ANOVA, *P*=0.0004). (**C**) OACs cultured for 7 days with 10 μM Oleu showed reduced expression of the mesenchymal markers CD105 and CD166, analyzed by flow cytometry. Student’s *t* test, *P*=0.0039 (CD105) and *P*=0.0022 (CD166), n=6 independent experiments. CD166 levels were also analyzed by western blot (n=3 independent experiments, Student’s *t* test, *P*=0.0046). (**D**) Downregulation of Cx43 increased Col2A1, detected by immunofluorescence in OACs treated with 10 μM oleuropein. Graphs represent the corrected total cell fluorescence (CTCF) of Cx43 and Col2a1 (n=4 independent experiments). Student’s *t* test, *P*<0.0001 (Cx43), and *P*=0.0007 (Col2a1). (**E**) mRNA levels of IL-1ß, IL-6, COX-2 and MMP-3 of OACs cultured in normal medium (UT) exposed to 10 μM oleuropein for 2 h. n=4–7 independent experiments. Student’s *t* test: *P*= 0.033 (IL-1ß), *P*<0.0001 (IL-6), *P*=0.1013 (COX-2), *P*=0.0466 (MMP-3). (**F**) IL-6 detected by ELISA when OACs were treated with oleuropein for 72 h (n=4 independent experiments, Student’s *t* test, *P*=0.0345). IL-6 (n=3 independent experiments) and COX-2 (n=4 independent experiments) protein levels detected by western-blot in OACs treated with 10 μM oleuropein for 72 h. Student’s *t* test, *P*=0.0193 (IL-6), *P*=0.0141 (COX-2). Data is expressed as mean±SD; *P<0.05, **P<0.01 and ***P<0.0001.

In concordance with these results, OACs’ redifferentiation for 7 days with oleuropein decreased Cx43 protein levels, but it did not affect its phosphorylation pattern (Figure 1F). However, we did not observe changes in the phosphorylation pattern when hMSCs or chondrocytes were treated with oleuropein (Figure 1F and Supplementary Figure 4A).

It is important to note that these results were obtained during differentiation of hMSCs under osteogenesis or chondrogenesis (Figure 1B–1E) and dedifferentiated OACs in normal and in chondrogenic medium (Figure 1A and 1F). However, the treatment of undifferentiated hMSCs cultured in basal growth medium with oleuropein/OE increased Cx43 levels (Supplementary Figure 4A and 4B) and GJIC (Supplementary Figure 4C), indicating that the effect of these olive derived polyphenols may be different depending on the cellular context.

### Downregulation of Cx43 activity by oleuropein downregulates Twist-1 and enhances redifferentiation of OACs

Oleuropein modulation of Cx43 significantly reduced GJIC in OACs (Figure 2A), but not in healthy chondrocytes (Figure 2B). The decrease in Cx43 and GJIC was correlated with a significant reduction in the levels of the stemness markers CD105 and CD166 (Figure 2C). This result was consistent with our previous observations, where CD105 and CD166 were reduced when Cx43 was downregulated or when OACs were redifferentiated. In fact, oleuropein effectively improved the OACs phenotype, detected by the increase of Col2A1 levels and the decrease of proinflammatory mediators and MMP-3 levels (Figure 2D, 2E). Oleuropein treatment reduced overall Cx43 positivity, increased Col2A1 levels (Figure 2D) and decreased interleukin 6 (IL-6), COX-2, IL-1ß and MMP-3 gene expression (Figure 2E) and protein levels (Figure 2F).

**Figure 3:**
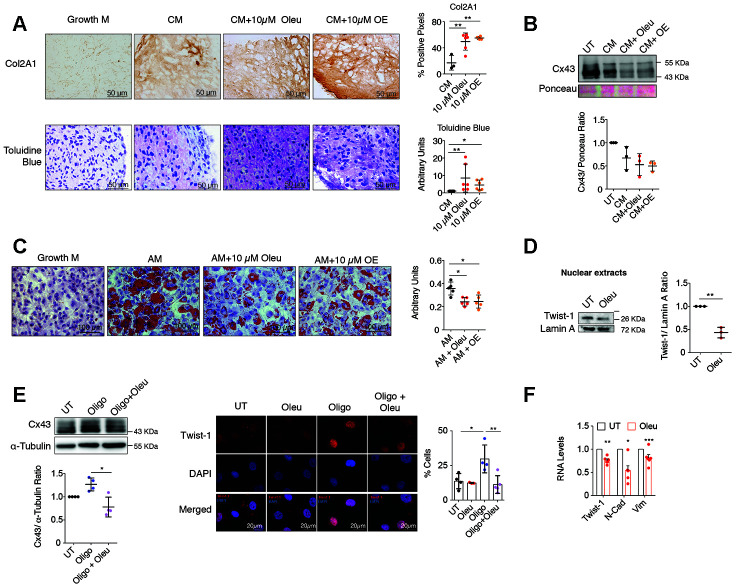
**Oleuropein treatment enhances chondrocyte redifferentiation.** (**A**) Immunohistochemistry of Col2A1 (4-6 independent experiments; one-way ANOVA, *P*=0.0019) and toluidine blue staining of proteoglycan subunits (n=6 independent experiments; one-way ANOVA, *P*=0.059) indicate significant enrichment in ECM components in OACs micromasses grown in 3D culture for 30 days in chondrogenic medium (CM) when supplemented with 10 μM oleuropein (Oleu) or OE. (**B**) Cx43 protein levels detected by western blot (and normalized to Ponceau staining) are reduced when OACs micromasses are exposed to CM supplemented with 10 μM oleuropein or OE for 21 days (n=3 independent experiments; one-way ANOVA, *P*=0.0328). (**C**) Oil red staining showing reduced OACs dedifferentiation upon exposure to Oleu or OE in adipogenic medium (n=5 independent experiments; one-way ANOVA, *P*=0.0001). (**D**) Nuclear levels of Twist-1 were decreased in OACs cultured with 10 μM oleuropein for 2 h. Lamin A was used as a loading control (n=3 independent experiments; Student’s *t* test, *P*=0.001). (**E**) Cx43 protein levels in primary OACs after 1-h treatment with oleuropein or oligomycin. Western blot represents n=4 independent experiments. Quantification is shown on the right (one-way ANOVA, *P*=0.0036). On the right, immunofluorescence for Twist-1 (red) in primary OACs treated with 5 μg/ml oligomycin and 10 μM oleuropein for 1 h. The graph represents the percentage of cells with Twist-1 nuclear localization (n=4 independent experiments; one-way ANOVA, *P*=0.0067). (**F**) The mRNA expression of the EMT markers Twist-1, N-Cadherin and Vimentin in OACs treated with 10 μM oleuropein for 2 h. Data were normalized to HPRT-1 levels. n= 5 independent experiments; Student’s *t* test: *P*<0.0001 (Twist-1), *P*= 0.0011 (N-Cad), *P*=0.0209 (Vim). Data is expressed as mean±SD; *P<0.05, **P<0.01 and ***P<0.0001.

Next, we sought to confirm whether oleuropein would target chondrocyte plasticity in 3D cultures. Treatment of OACs grown as a 3D culture in chondrogenic medium with oleuropein increased the deposition of proteoglycans and Col2A1 (Figure 3A), improving the ECM structure by decreasing Cx43 levels (Figure 3B).

To explore the effect of oleuropein on cell plasticity, OACs were grown in adipogenic medium supplemented with oleuropein, which significantly decreased their adipogenic differentiation (Figure 3C). However, oleuropein promoted osteogenesis when OACs were grown in osteogenic medium supplemented with 10 μM of oleuropein (Supplementary Figure 5).

Upregulation of Cx43 in OA involves dedifferentiation via chondrocyte-to-mesenchymal transition by Twist-1 activation, which was also reported in OA cartilage [2, 70, 71]. OACs were treated with the arthritic insult oligomycin, which induces cellular ROS production and cartilage degradation [72, 73]. After oleuropein treatment, OACs showed a significant reduction in the nuclear localization of Twist-1 (Figure 3D). Nuclear localization of Twist-1 in the presence of oligomycin was attenuated when Cx43 protein levels were reduced by oleuropein treatment of OACs (Figure 3E). In addition, oleuropein treatment in OACs reduced the expression of the mesenchymal and EMT markers N-cadherin and vimentin (Figure 3F).

### Oleuropein modulates Cx43 gene promoter activity

**Figure 4:**
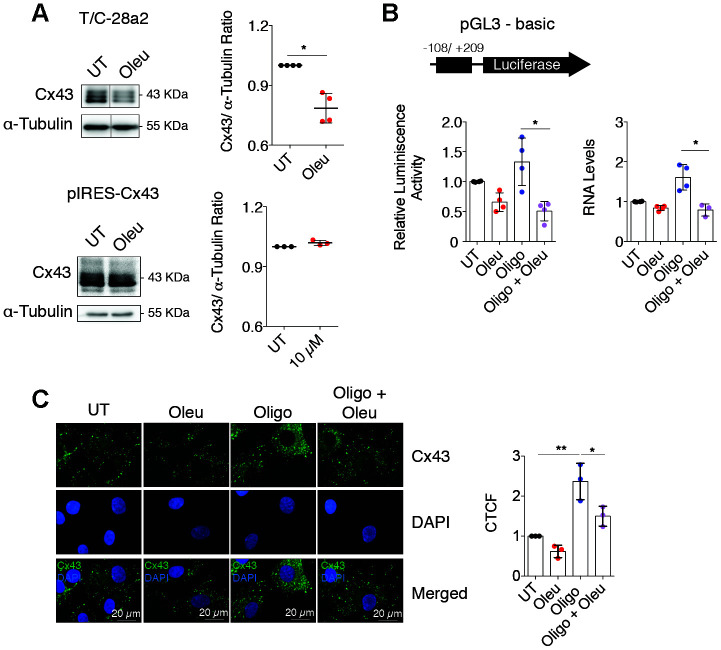
**Oleuropein modulates the Cx43 promoter activity in chondrocytes.** (**A**) Treatment with 10 μM oleuropein for 2 h decreases Cx43 protein levels in T/C-28a2 cells (n=4 independent experiments, Student’s *t* test, *P*=0.0012), but this effect was not observed in the same cell line overexpressing Cx43 (pIRES-Cx43)(n=3 independent experiments, Student’s *t* test, *P*=0.0624). (**B**) Luciferase reporter assay indicating that oleuropein inhibits Cx43 promoter activity. The graphs indicate the normalized luminescence activity in the T/C-28a2 transfected with a pGL3-basic plasmid containing 300 base pairs of Cx43 promoter ligated to the luciferase gene. Cells were cultured in DMEM with 10% FBS (UT) and with 5 μg/ml oligomycin or 10 μM oleuropein for 1 h as indicated (n=4 independent experiments; one-way ANOVA, *P*=0.0012). On the right, Cx43 gene expression under 5 μg/ml oligomycin and 10 μM oleuropein treatment in OACs treated for 1 h (n=4 independent experiments; one-way ANOVA, *P*=0.0002). Data were normalized to HPRT-1 levels. (**C**) Immunofluorescence assays of Cx43 in OACs treated with 10 μM oleuropein or 5 μg/ml oligomycin for 1 h. Data were normalized to the untreated condition (n=3 independent experiments; one-way ANOVA, *P*<0.0001). Data is expressed as mean±SD; *P<0.05, **P<0.01 and ***P<0.0001.

In chondrocytes, decreased Cx43 protein levels were detected when OACs and the T/C-28a2 cell line were treated with oleuropein (Figures 1A and 4A). However, these changes were not evident in treated Cx43-overexpressing T/C-28a2 chondrocytes (Figure 4A, bottom), suggesting that oleuropein may affect Cx43 gene promoter activity rather than protein stability. We thus measured whether oleuropein affected the activity of the Cx43 gene promoter using a real-time reporter system. T/C-28a2 chondrocytes were transfected with a firefly luciferase reporter vector containing the regulatory regions of the Cx43 promoter and incubated for 1 h with oleuropein and the mitochondrial inhibitor oligomycin, which enhanced Cx43 gene expression (Figures 3E, 4B). Cx43 promoter activity decreased after oleuropein treatment (Figure 4B), while oligomycin increased the luminescence signal, and the effect of oligomycin was significantly attenuated in the presence of oleuropein (Figure 4B), which was correlated with Cx43 gene expression (Figure 4B). Luminescence signal strongly correlated with the effects of oleuropein and oligomycin on protein levels (Figures 3E and 4C). These results indicate that oleuropein affects Cx43 gene promoter activity, thus downregulating Cx43 protein levels (Figure 1A and Figure 4A–4C) and GJIC in OACs (Figure 2B).

**Oleuropein enhances elimination of senescent cells**

**Figure 5:**
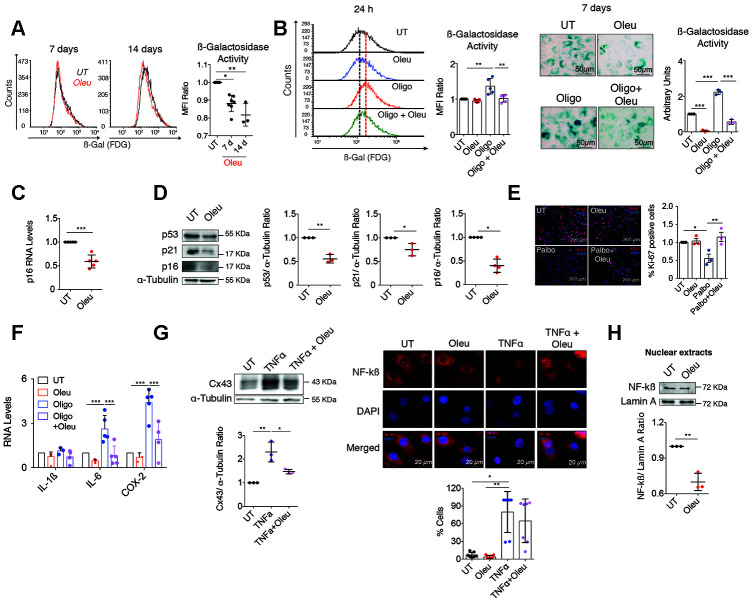
**Cx43 downregulation by oleuropein decreased chondrocyte senescence.** (**A**) SA-βGal activity detected by flow cytometry in OACs treated with 10 μM oleuropein (Oleu) for 7 and 14 days (n=3–7 independent experiments; one-way ANOVA, *P*<0.0001). (**B**) The graphs show the comparative analysis of SA-βGal activity measured by flow cytometry of OACs exposed for 24 h to 10 μM oleuropein or 5 μg/ml oligomycin as indicated (n=5 independent experiments; one-way ANOVA, *P*=0.0003). On the right, SA-βGal activity determined by X-Gal cleavage and cell staining (blue), evaluated by microscopy in OACs treated for 7 days with 10 μM oleuropein or 5 μg/ml oligomycin (n=3 independent experiments; one-way ANOVA, *P*<0.0001). (**C**) p16 mRNA expression of OACs treated with 10 μM oleuropein for 2 h. Data were normalized to HPRT-1 levels (n=5 independent experiments; Student’s *t* test, *P*=0.0002). (**D**) Western blot of p53 (n=3 independent experiments), p21 (n=3 independent experiments) and p16 (n=4 independent experiments) in OACs treated with 10 μM oleuropein for 2 h. α-tubulin was used as a loading control. Student’s *t* test, *P*=0.001 (p53), *P*=0.0278 (p21), *P*=0.0286 (p16). (**E**) Cell proliferation evaluated by immunofluorescence of Ki-67 in T/C-28a2 chondrocytes treated with 10 μM palbociclib and/or 10 μM oleuropein for 24 h. Images represent n= 3 independent experiments. One-way ANOVA, *P*=0.0434 (UT vs Palbo); *P*=0.0096 (Palbo vs Palbo+Oleu). (**F**) Downregulation of Cx43 by oleuropein attenuates IL-6 and COX-2 upregulation when OACs are exposed to oligomycin for 1 h (n=3–9 independent experiments; one-way ANOVA). (**G**) Western blot (n=3 independent experiments) shows the effect of 10 μM oleuropein and 10 ng/mL TNFα treatments (for 1 h) on Cx43 protein levels in OACs (one-way ANOVA, *P*=0.0018). On the right, NF-κB detected by immunofluorescence in OACs treated with 10 ng/mL TNFα for 1 h. This effect is partially abolished by 1-h 10 μM oleuropein treatment. The graph represents the cell percentage with nuclear NF-κB staining (n=7 independent experiments; one-way ANOVA, *P*=0.0055). (**H**) Nuclear levels of NF-kß in OACs cultured with 10 μM oleuropein for 2 h. Lamin A was used as a loading control (n=3 independent experiments; Student’s *t* test, *P*=0.0021). Data is expressed as mean±SD; *P<0.05, **P<0.01 and ***P<0.0001.

Senescent cells through their secretory activity (SASP) can promote dedifferentiation and reprogramming in neighboring cells in the context of tissue injury [74]. OACs treated with 10 μM oleuropein for 7/14 days in growth medium showed a significant reduction of senescent cells accumulated after 5 days of primary culture (Figure 5A). Consistent with these models, Cx43 upregulation due to the oligomycin insult significantly contributed to increase cellular senescence in OACs accumulated after 5 days in monolayer (Figure 5B, left), whereas co-treatment with oleuropein significantly halted the accumulation of senescent cells after 24 h (Figure 5B, left) and 7 days under treatment (Figure 5B, right). Interestingly, increased SA-βGal activity was detected when the T/C-28a2 chondrocytes were treated with bleomycin to induce cellular senescence for 24 h, while the exposure to oleuropein for 24 h reduced the number of senescent cells (Supplementary Figure 6B). In accordance with these results, treatment of OACs with oleuropein led to decreased levels of the senescence biomarkers p16^INK4A^ (Figure 5C) and p53/p21 (Figure 5D). Furthermore, the proliferative arrest observed after the treatment of T/C-28a2 healthy chondrocytes with the Cyclin-Dependent Kinase 4/6 inhibitor palbociclib to induce senescence was partially inhibited by the co-treatment with oleuropein (Figure 5E). Oleuropein reduced the accumulation of senescent cells and attenuated the oligomycin-induced SASP secretion detected by IL-6, COX-2 and IL-1ß gene expression in chondrocytes (Figure 5F). The SASP, including IL-6 gene expression, can be activated by NF-κB [75]. Oleuropein protected from the increase of Cx43 under TNFα treatment (Figure 5G, left), and NF-κB (p65) activation by TNFα in OACs was diminished when cells were exposed to oleuropein for 1 h (Figure 5G). Further, NF-κB nuclear translocation was partially abolished in OACs treated with oleuropein for only 2 h (Figure 5H).

**Figure 6:**
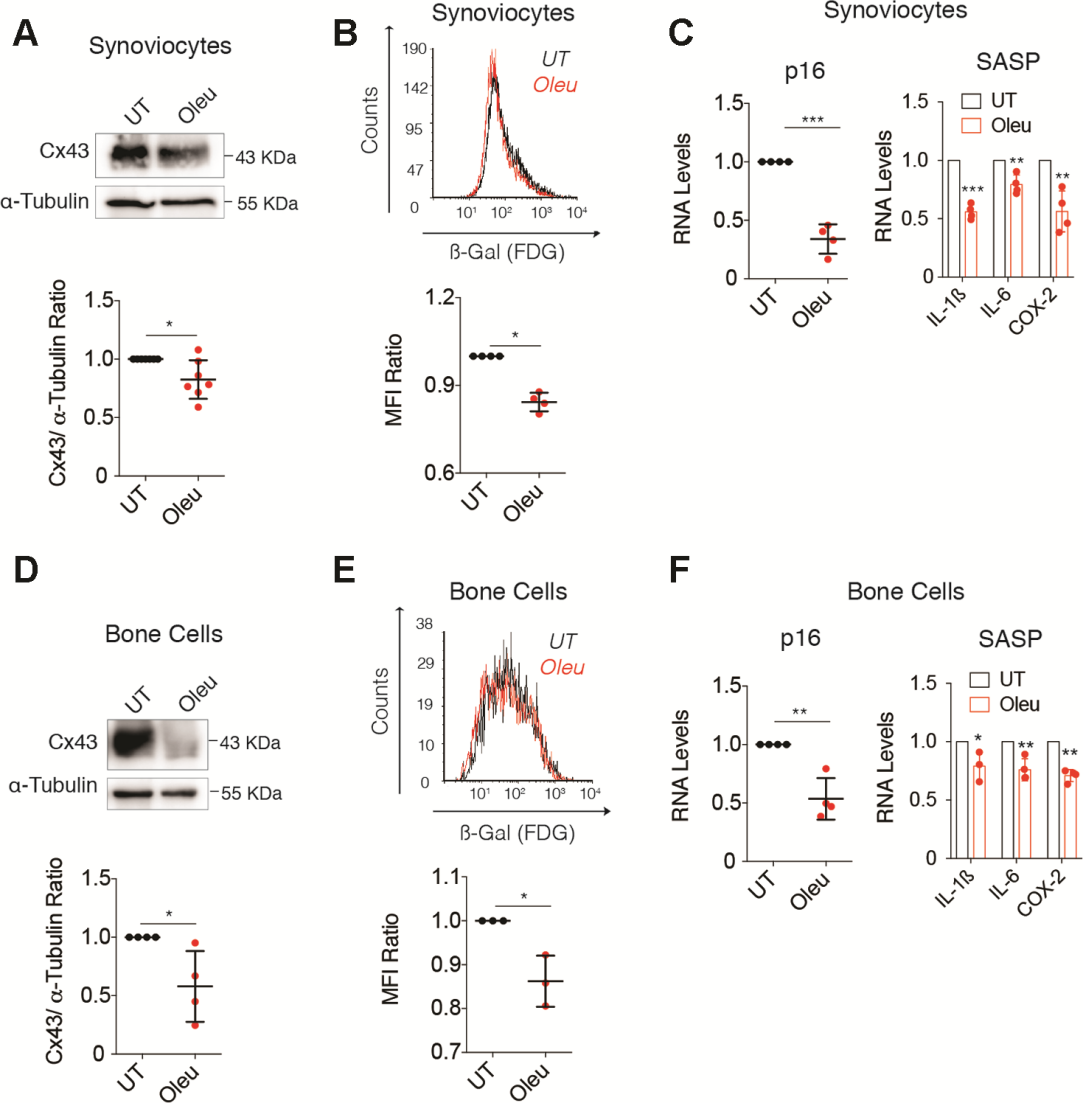
**Oleuropein treatment decreased cellular senescence in synoviocytes and bone cells isolated from patients.** (**A**) Cx43 protein levels analyzed by western blot in synoviocytes treated with 10 μM oleuropein for 2 h (n=7 independent experiments, *P*=0.0313). (**B**) Treatment of synoviocytes with 10 μM of oleuropein for 7 days detected by SA-βGal activity (n=4 independent experiments, *P*<0.0001). (**C**) p16 mRNA levels of synoviocytes treated with 10 μM oleuropein for 2 h. Data were normalized to HPRT-1 levels (n= 4 independent experiments, *P*<0.0001). On the right, mRNA levels of IL-1ß, IL-6 and COX-2 of synoviocytes cultured in normal medium (DMEM 10% FBS) and exposed to 10 μM oleuropein for 2 h. Data were normalized to HPRT-1 levels. N=4 independent experiments, *P*<0.0001 (IL-1ß), *P*=0.0024 (IL-6), *P*=0.0025 (COX-2). (**D**) Cx43 protein levels analyzed by western blot in bone cells treated with 10 μM oleuropein for 2 h (n=4 independent experiments, *P*=0.0319). (**E**) 10 μM of oleuropein treatment for 7 days reduces senescence levels in bone cells as detected by SA-βGal and flow cytometry (n=3 independent experiments, *P*=0.0149). (**F**) p16 mRNA expression of bone cells treated with 10 μM oleuropein for 2 h. Data were normalized to HPRT-1 levels (n= 4 independent experiments, *P*=0.002). On the right, mRNA levels of IL-1ß, IL-6 and COX-2 of bone cells cultured in normal medium (DMEM 10% FBS) exposed to 10 μM oleuropein for 2 h. Data were normalized to HPRT-1 levels. N=3-4 independent experiments. *P*=0.0463 (IL-1ß), *P*=0.0077 (IL-6), *P*=0.0002 (COX-2). Data is expressed as mean±SD, Student’s *t* test; *P<0.05, **P<0.01 and ***P<0.0001.

To further test the senolytic activity of oleuropein, SC and BC from OA patients were treated with oleuropein (Figure 6). We observed decreased Cx43 protein levels after oleuropein exposure (Figure 6A and 6D), together with a significant reduction in senescent cells accumulated after 5 days in primary culture (Figure 6B and 6E), confirmed by p16^INK4A^ gene expression and the synthesis of the SASP factors IL-1ß, COX-2 and IL-6 (Figure 6C and 6F).

## DISCUSSION

Previous data from our group demonstrate that Cx43 downregulation improves the chondrocyte phenotype, protecting chondrocytes from dedifferentiation and senescence [2]. Although oleuropein and olive-based diets were reported to protect from OA progression [76, 77], there was no solid evidence about its underlying molecular mechanisms. In our study, we show that oleuropein modulates Cx43 gene promoter activity, reducing Cx43 and GJIC in OACs. Indeed, our data indicate that Cx43 downregulation by oleuropein in OACs improves cell phenotype by protecting chondrocytes from Twist-1 activation and from accumulation of senescent cells (Figures 2, 3, 5 and 6). This is the first study that demonstrated one of the potential mechanisms of oleuropein in OACs, SC and BC (Figures 5 and 6) from patients, and in hMSCs (Figure 1), with relevant applications in regenerative medicine.

Our results show that the effects of oleuropein are often equal or even smaller than those of the olive extract, suggesting that other compounds may synergize with oleuropein activity in chondrocytes [78]. On the other hand, the treatment of hMSCs with oleuropein or OE during differentiation leads to downregulation of Cx43 and GJIC, enhancing osteogenesis and chondrogenesis, but reducing adipogenesis. This differential sensitivity of hMSCs to oleuropein and OE may have potential applications in preventive and regenerative medicine in other bone and cartilage disorders.

Our data show decreased Cx43 and GJIC levels in the presence of oleuropein in OACs and in differentiating hMSCs, but increased Cx43 and GJIC levels in undifferentiated hMSCs, suggesting that the effect of this molecule on GJIC depends on its effect on Cx43 levels (or subcellular localization). In fact, phosphorylation of Cx43 affects protein stability and GJIC activity [79] and we have detected changes in Cx43 phosphorylation pattern during hMSCs differentiation but not under oleuropein treatment indicating that oleuropein affects Cx43 levels more than gap junction plaque activity or modulation. We cannot therefore discard that the effect of oleuropein may also depend on channel-independent activities, which involve the signaling hub’s ability of Cx43 to recruit proteins to the membrane [34, 80, 81] or its ability to control gene transcription [82]. It is important to note that oleuropein may have other targets that may contribute to the drug effect. Despite this limitation and based on our results, we expect that the effect of oleuropein occurs at least partially through Cx43 modulation. Here, we show that oleuropein restores chondrocyte phenotype detected by reduced levels of the stem-markers CD105, CD166, N-cad and vimentin (Figure 2C, 3F). The effect of oleuropein in chondrocyte plasticity correlated with activation of redifferentiation via downregulation of Cx43 and Twist-1 (Figure 3D–3F), leading to increased levels of proteoglycans and Col2A1 together with decreased levels of inflammatory cytokines and metalloproteinases (Figures 2D–2G, and 3A).

In cell culture and in cartilage, OACs undergo dedifferentiation and senescence [2, 18, 20]. Elimination of senescent cells *in vivo* using the senolytic drug UBX0101 has been demonstrated to improve cartilage regeneration after articular joint injury in mice [1]. Here we show that oleuropein reduces cellular senescence in OACs, SC and BC and protects from accumulation of senescent cells under an arthritic insult (Figures 5A, 5B, 6B and 6F). NF-kB has been shown to regulate the inflammatory components of the SASP, together with other factors [83, 84]. In this study the reduction of senescence is accompanied by reduced NF-kB activity, and therefore reduced synthesis of SASP (Figure 5F). Notably, these components enhance inflammation, senescence and activate dedifferentiation and cellular reprogramming of nearby non-senescent cells (e.g. via IL-6) [74], contributing to the stem-like state of chondrocytes in OA and to the accumulation of senescent cells. Accordingly, we have previously reported that upregulation of Cx43 leads to p53/p16 upregulation and senescence [2]. Using the T/C-28a2 cells with a Cx43 overexpression vector and a CRISPR/Cas9-mediated heterozygous Cx43 gene knockdown cell line we have demonstrated that Cx43 is an upstream effector of both senescence (involving p53 and p16 pathways) and NF-kB activation [2].

**Figure 7:**
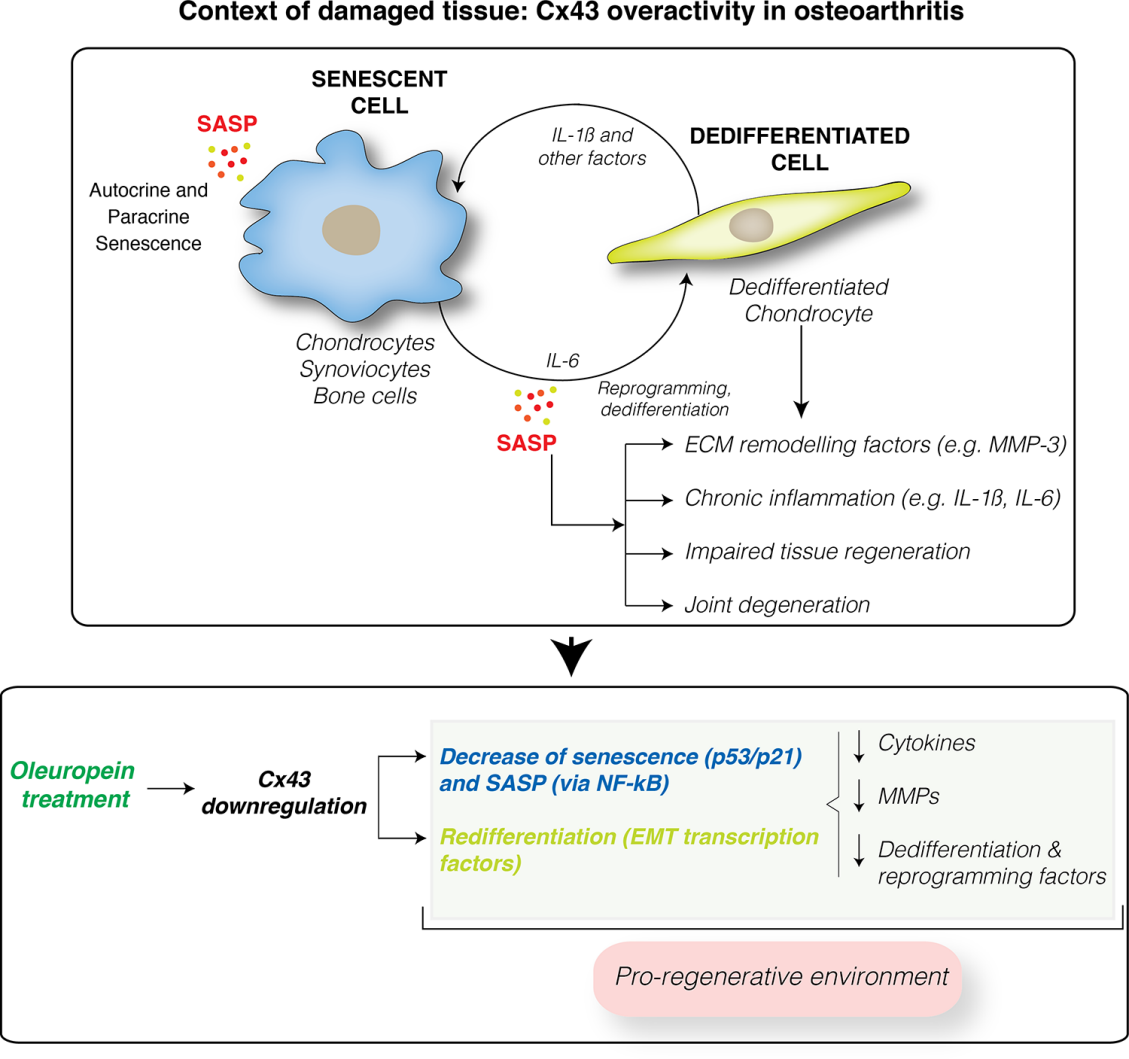
**Cx43 overactivity contributes to disease progression.** Cx43 overexpression leads to accumulation of dedifferentiated and senescent cells involved in disease progression in OA patients. These phenotypic changes results in the synthesis of ECM remodeling factors involved in tissue degradation (MMPs) and proinflammatory factors, such as IL-1ß and IL-6, which facilitate the dedifferentiation and reprogramming of neighboring cells. These factors may further spread senescence and dedifferentiation to surrounding tissues contributing to joint degeneration. Downregulation of Cx43 by oleuropein treatment contributes to the elimination of senescent cells and redifferentiation of osteoarthritic chondrocytes into fully differentiated cells, able to support the ECM composition and restoring the regenerative capacity of the tissue. However, oleuropein may have other targets that may contribute to the drug effect. In addition, oleuropein treatment might improve the effectiveness of stem cell therapy, by promoting chondrogenic and osteogenic differentiation, and by inhibiting adipogenesis.

Cellular reprogramming, dedifferentiation via EMT and senescence play active roles during tissue regeneration [85, 86]. Accumulation of dedifferentiated (stem-like cells) and senescent cells leads to impaired tissue regeneration and fibrosis with loss of tissue function [87] (Figure 7). Understanding and manipulating the complex Cx43 signaling would expand our opportunities for modulating wound-healing related disorders such as OA. So far, our results indicate that molecules that reduce Cx43 levels in OA, including oleuropein, will potentially contribute to create a regeneration-permissive environment in OA patients.

Our preliminary study is the first to demonstrate the effect of this polyphenol on Cx43 activity and senescence. Besides, modulation of Cx43 by oleuropein shifts the hMSCs differentiation capacity towards osteogenic and chondrogenic lineages, while decreasing adipogenic differentiation. It is important to remark that aging has been reported to reduce the reparative potential of MSCs [31]. Hence, the use of small polyphenols such as oleuropein may be useful in cell therapy approaches that aim to promote cartilage and bone regeneration, as the osteogenesis/adipogenesis switch has been associated with different bone disorders [88, 89]. Altogether, these findings indicate that oleuropein controls Cx43 gene expression and acts as a senolytic drug that may serve as a potential agent to improve both the effectiveness of stem cell therapy and cartilage and joint regeneration in patients to stop OA progression. Thus, the implementation of therapies based on Cx43 modulators or innovative approaches based on functional foods enriched in olive extracts may improve joint functions and quality of life for OA patients by reducing senescence and inducing a pro-regenerative environment.

## MATERIALS AND METHODS

### Tissue collection, processing and cell culture

Collection and processing of cartilage from human knees and femoral heads from adult donors undergoing joint surgery were performed as previously described [10]. Briefly, primary chondrocytes were isolated from cartilage by mechanical (dicing) and enzymatic digestion, with an incubation with 0.5 mg/mL of trypsin-EDTA (Gibco, Thermo Fisher Scientific) for 10 min at 37ºC, followed by an overnight incubation at 37ºC with a 2 mg/mL collagenase IV solution, with shaking. Finally, digested cartilage was filtered through a 100 μm strainer and chondrocytes were seeded onto 100 mm dish plates (Corning, Sigma-Aldrich). The study was conducted with the approval of the institutional ethics committee (C.0003333, 2012/094 & 2015/029) after the acquisition of signed informed consents. We have collected cartilage samples (1 sample per patient) from a total amount of 22 donors (14 OA donors and 8 healthy donors) from hip and knee joints with a mean age of 72.1±4,7 years (50% women and 50% men) within OA donors, and a mean age of 81.62±2.9 years (75% women and 25% men) within healthy donors. Cartilage samples from healthy donors were obtained after knee or hip fracture with no history of joint disease. All samples (healthy and OA) were analyzed by histological staining and classified following the American College of Rheumatology (ACR) recommendations and clinical classification criteria for OA. The chondrocyte cell line T/C-28a2 was kindly donated by Dr. Goldring (Hospital for Special Surgery; New York, USA). Primary chondrocytes and T/C-28a2 cells were cultured in Dulbecco’s Modified Eagle’s medium (DMEM) supplemented with 100 U/mL penicillin, 100 μg/mL streptomycin and 10% foetal bovine serum (FBS). Human mesenchymal stem cells (hMSCs) were obtained with signed informed consent from 4 bone marrow donors (*Hospital Universitario Reina Sofía*; Córdoba, Spain) and from subcutaneous inguinal fat from 2 healthy individuals (*Hospital Universitario Ramón y Cajal*; Madrid, Spain). hMSCs were cultured in MesenPRO RS^TM^ Medium supplemented with 100 U/ml penicillin and 100 μg/ml streptomycin. Synoviocytes (cultured in RPMI 10%FBS supplemented with 1% insulin-transferrin-selenium, 100 U/mL penicillin and 100 μg/mL streptomycin) and bone cells (cultured in DMEM supplemented with 100 U/mL penicillin, 100 μg/mL streptomycin and 20% inactivated FBS) from OA patients were donated by Raquel Largo (IIS-Fundación Jiménez Díaz; Madrid, Spain), after signed informed consent. hMSCs, primary chondrocytes, T/C-28a2 cells, BC and SC at 70-80% confluence were treated with oleuropein (Extrasynthese #0204; Lyon, France) or an olive extract (OE) containing 41.5% of oleuropein, donated by the Clinical Management Unit of Endocrinology and Nutrition (IMIBIC, Córdoba, Spain). OE was dissolved in culture medium to a stock concentration equivalent to 100 μM oleuropein. Both compounds were dissolved in the cell culture medium and added to the cells for short-term (1–2 h) or long-term (7–14—21 days; each 48 hours) treatments. Bone cells and synoviocytes were treated with oleuropein 10 μM for 2 h at a 70% confluence. For cell treatments, chondrocytes at a 70-80% confluence were treated for 1 h with either 5 μg/ml oligomycin (Sigma-Aldrich) or 10 ng/mL TNFα (Immunotools). In addition, these cells were also treated simultaneously for 1 h with the combination of oligomycin or TNFα with 10 μM oleuropein. Cell proliferation arrest in T/C-28a2 healthy chondrocytes was performed by a 24h-treatment with 10 μM of Palbociclib (APExBIO) or the combination of Palbociclib and 10 μM oleuropein.

### Cell viability assay

Cells at a 75% confluence were treated with 0.1 μM, 1 μM, 10 μM and 10 mM oleuropein for 17 h. Drug cytotoxicity was evaluated by the colorimetric MTT assay (Cell Proliferation Kit I, Roche) with a NanoQuant microplate reader (Tecan Trading AG) at 570 nm.

### Adipogenic differentiation

Adipogenic differentiation was performed as previously described [2]. Cells were incubated with adipogenic medium (hMSC Adipogenic Differentiation Bullekit^TM^, Lonza) for 21 days, with the addition of oleuropein or OE. Adipogenic differentiation was evaluated by the RNA expression of PPARγ and by oil red O staining.

### Osteogenic differentiation

For osteogenic differentiation, cells were differentiated for 21 days with osteogenic medium (StemPro® Osteogenesis Differentiation Kit, Gibco, Thermo Fisher Scientific), with the addition of oleuropein or OE. Osteogenesis was evaluated by the RNA expression of OSTCN and by alizarin red S staining.

### Chondrogenic differentiation

Chondrogenesis was performed in OACs and hMSCs as cell micromasses (3D) or as a monolayer culture. For the 3D culture, 500000 cells were seeded in non-adherent conic tubes and centrifuged at 500 x*g* for 10 min, resulting in a pellet formation, and cultured in chondrogenic medium (CM; StemPro® Chondrogenesis Differentiation Kit, Gibco, Thermo Fisher Scientific) supplemented with oleuropein or OE for 30 days. For the monolayer culture, cells were differentiated in CM for 7/14 days with oleuropein/OE addition. Chondrogenesis was evaluated by proteoglycans detection (toluidine blue staining), Col2A1 immunohistochemistry and by ACAN mRNA expression.

### Scrape loading/dye transfer (SL/DT) assay

Confluent cells were treated with oleuropein or OE for 2 h. Next, a 0.4% (w/v) solution of lucifer yellow (LY) (Cell Projects Ltd©) was loaded and two distant scrapes were made across the culture plate. In order to evaluate GJIC, a score was calculated as the ratio of non-damage positive cells for LY to the damaged cells [2, 10].

### GJ connectivity by flow cytometry

Equal numbers of cells were incubated for 1 h at 37 ºC with either 1 μM of the membrane dye DiI (Invitrogen, Thermo Fisher Scientific) or 1 μM calcein-AM (Invitrogen, Thermo Fisher Scientific), a cell-permeable dye that is retained in the cytosol and can be transferred by GJs. Then, cells were washed with PBS and co-cultured for 2 h in a 1:4 ratio (calcein-donors:DiI-recipient cells). The percentage of DiI^+^Calc^+^ cells was analysed by flow cytometry.

### Western blot

Protein analysis was performed as previously described [2, 10]. The following primary antibodies were used: α-tubulin (Sigma-Aldrich, T9026), Cx43 (Sigma-Aldrich, C6219), Twist-1 (Santa Cruz Biotechnology, sc-81417), CD166 (Santa Cruz Biotechnology, sc-74558), p16^INK4A^ (Abcam, ab108349), p21 (Santa Cruz Biotechnology, sc-6246), p53 (Santa Cruz Biotechnology, sc-126), NF-κB (Santa Cruz Biotechnology, sc-8008), lamin A (Santa Cruz Biotechnology, sc-20680), IL-6 (Sigma-Aldrich, SAB1403971) and COX-2 (Santa Cruz Biotechnology, sc-166475).

### Enzyme-linked immunosorbent assay (ELISA)

Supernatants were collected after a 72 h-treatment with oleuropein 10 μM, and diluted in a 1:8 ratio prior to the measurement. Samples were incubated for 2 h at room temperature, and IL-6 was detected using the quantitative sandwich human IL-6 immunoassay (hIL-6-EIA-20, MagTag GmbH). Samples were analyzed at 450 nm in a TECAN plate reader.

### Antigen expression analysis by flow cytometry

Paraformaldehyde-fixed cells were incubated with phycoerythrin (PE)-conjugated anti-human CD105 (Immunostep, 105PE-100T) or allophycocyanin (APC)-conjugated anti-human CD166 (Immunostep, 1399990314), for 30 min at 4°C, as previously reported [2] (Supplementary Table 1). Intracellular Cx43 protein was detected in paraformaldehyde-fixed cells after permeabilization with methanol for 30 min at 4ºC. Finally, cells were incubated with APC-conjugated anti-human Cx43 antibody (R&D Systems, FAB7737A) for 30 min at 4°C.

### Flow cytometry analysis

20.000 events were collected on a FACSCalibur^TM^ (Becton Dickinson) flow cytometer with the CellQuest^TM^ Pro software. Cell debris was discriminated by the forward scatter (FSC) and side scatter (SSC) properties of the cells. Data were analyzed with FCS Express 6 Flow software (De Novo Software). The level of positive staining was expressed as the median fluorescence intensity (MFI), with unlabelled cells as negative controls. Gates were placed based on single-labelled controls and by establishing 0.1% as the cut-off point.

### Senescence-associated β-galactosidase activity

Flow cytometry analysis of SA-βGal activity with the fluorogenic β-galactosidase substrate fluorescein di-β-D-galactopyranoside (FDG; Invitrogen, Thermo Fisher Scientific) was performed as previously described [2]. Briefly, cells were harvested and incubated with pre-warmed 2 mM FDG for 3 min at 37 ºC and fluorescein positivity was analyzed on a BD FACSCalibur^TM^ (Becton Dickinson) flow cytometer. SA-βGal activity was also measured with the Senescence Cells Histochemical Staining Kit (Sigma-Aldrich) according to the manufacturer’s protocol and analyzed after 16 h of staining.

### Immunofluorescence and immunohistochemistry

Immunofluorescence and immunohistochemistry assays were performed as previously described [2, 10]. The following primary antibodies were used: anti-Cx43 (Sigma-Aldrich, C6129), anti-collagen II (Invitrogen, Thermo Fisher Scientific, MA5-12789), anti-Ki-67 (BD, 550609), anti-Twist-1 (sc-81417) and anti-NF-κB (sc-8008) from Santa Cruz Biotechnology. Goat anti-rabbit FITC-conjugated (F-2765, Invitrogen, Thermo Fisher Scientific) and goat anti-mouse Alexa 594-conjugated (A-11032, Invitrogen, Thermo Fisher Scientific) secondary antibodies were used.

### Quantitative PCR

TRIzol^TM^ reagent (Invitrogen, Thermo Fisher Scientific) was used to isolate total RNA, according to the manufacturer's instructions. 1 μg of total RNA per reaction was used to synthesize cDNA with the SuperScript® VILO™ cDNA Synthesis Kit (Invitrogen, Thermo Fisher Scientific). Quantitative PCR was performed as previously described [2, 10]. Primers are listed in Supplementary Table 2.

### Cell transfection

Cx43 was overexpressed, as previously described [2], in the T/C-28a2 chondrocyte cell after transfection with a pIRESpuro2 plasmid construct (Clontech) containing the human Cx43 sequence, kindly provided by Arantxa Tabernero (INCL, University of Salamanca, Spain). Electroporation was performed with the Amaxa® Cell Line Nucleofector® Kit V (Lonza) in a Nucleofector^TM^ 2b device (Lonza) following the manufacturer’s instructions.

### Luciferase reporter assay

A DNA construct containing the upstream 300 bp-regulatory sequence of the human Cx43 promoter (–108, +279, relative to the human Cx43 transcription start site) in a pGL3-Basic vector was kindly donated by Dr. Mustapha Kandouz (Wayne State University, USA). The T/C-28a2 chondrocyte cell line was transfected with the Amaxa® Cell Line Nucleofector® Kit V in a Nucleofector^TM^ 2b device. After 24 h, cells were treated with 10 μM oleuropein and/or 5 μg/ml of oligomycin (Sigma-Aldrich) for 1 h. For the luminescence analysis, the Firefly Luciferase Assay Kit from Biotium was used according to the manufacturer’s instructions, and normalized to the total protein content.

### Statistical analysis

Statistical analysis was performed with GraphPad Prism software (version 7.0a). Data of the biological and/or technical replicates are represented as mean and SD. Two-tailed Unpaired Student’s *t* test was used to estimate statistically significant differences between two groups. The estimation of the difference of the means among groups was compared by one-way ANOVA with Bonferroni correction. *P* values were provided, with *P*<0.05 considered as statistically significant. *P<0.05, **P<0.01 and ***P<0.0001.

## Supplementary Material

Supplementary Figures

Supplementary Tables
