# Supplementary material for: Senolytic activity of small molecular polyphenols from olive restores chondrocyte redifferentiation and promotes a pro-regenerative environment in osteoarthritis
Source: Aging (Albany NY). 2020 Aug 3;12(16):15882–905. doi: 10.18632/aging.103801 (PMC7485729; doi:10.18632/aging.103801)
Supplement: Supplementary Figures [file aging-12-103801-s002..pdf]

# SUPPLEMENTARY FIGURES

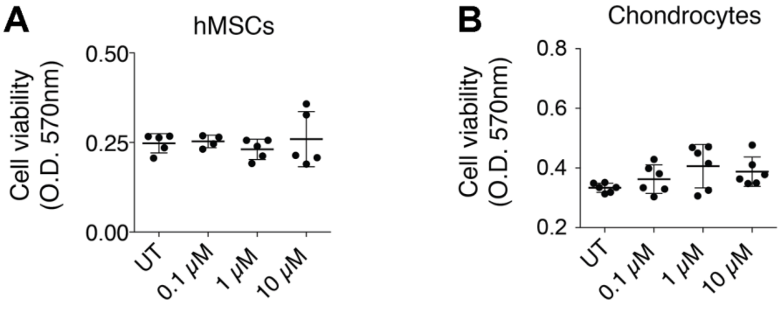


**Supplementary Figure 1.** Cell viability measured by MTT assay of (**A**) hMSCs from both bone marrow or subcutaneous inguinal fat (n=5 independent experiments, *P*=0.1065) and (**B**) chondrocytes (n=6 independent experiments, *P*=0.7871) exposed to different concentrations of oleuropein (Oleu) for 17 h. Data is expressed as mean±SD, one-way ANOVA. **P*<0.05, ***P*<0.01 and ****P*<0.0001.

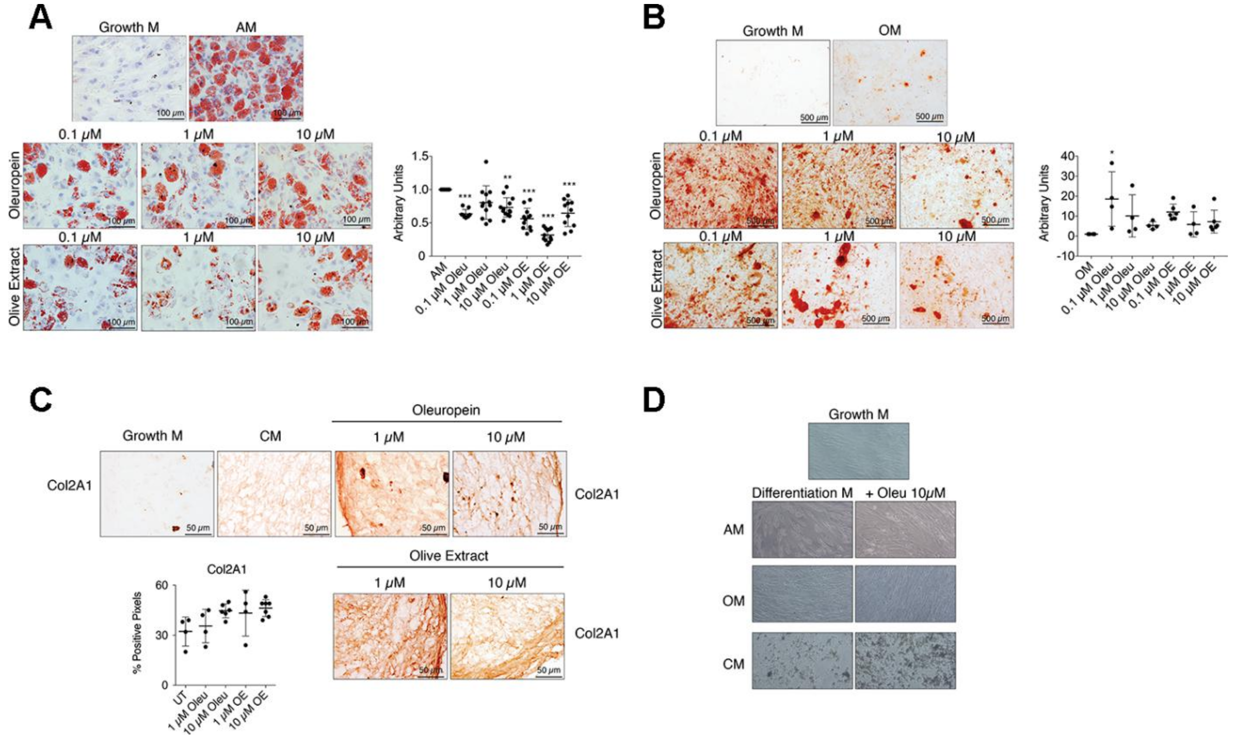


**Supplementary Figure 2.** (**A**) Adipogenic differentiation of hMSCs exposed to different concentrations of oleuropein (Oleu) or OE. Cells were stained with oil red O for lipid evaluation. The graph represents the ratio of cells with lipid deposits to the total number of cells and was normalized to hMSCs cultured in adipogenic medium (AM) (n=3-9 independent experiments, *P*<0.0001). (**B**) Osteogenic differentiation of hMSCs treated with oleuropein or OE for 21 days. Alizarin red staining was performed to evaluate calcium deposits. Quantification was normalized to hMSCs cultured in osteogenic medium (OM) (n=4-6 independent experiments, *P*=0.0166). (**C**) Chondrogenic differentiation of hMSCs for 30 days exposed to different concentrations of oleuropein or OE. Images represent Col2A1 immunohistochemistry (n=4-7 independent experiments, *P*=0.0609). (**D**) Images showing the phenotypes of hMSCs differentiated for 14 days with or without oleuropein. AM (adipogenic differentiation), OM (osteogenesis) and CM (chondrogenesis). Data is expressed as mean±SD, one-way ANOVA, **P*<0.05, ***P*<0.01 and ****P*<0.0001.

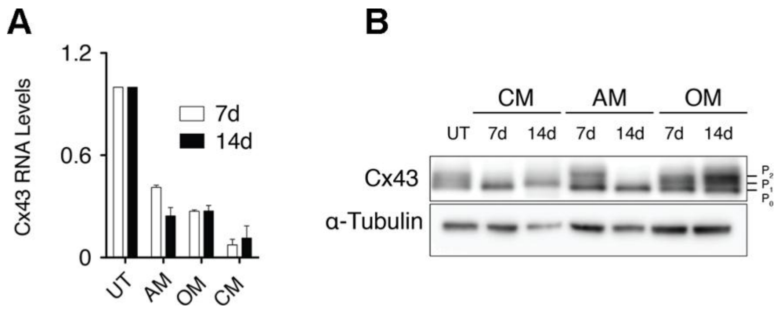


**Supplementary Figure 3.** (**A**) Cx43 levels analyzed by RT-qPCR of hMSCs differentiated with adipogenic (AM), osteogenic (OM) or chondrogenic (CM) medium for 7 and 14 days (n=2-3 independent experiments; data were normalized to HPRT-1 levels and are shown as mean±SD). (**B**) Comparative Cx43 protein levels of hMSCs cultured in chondrogenic (CM), adipogenic (AM) and osteogenic medium (OM) for 7 and 14 days. α-tubulin was used as a loading control.

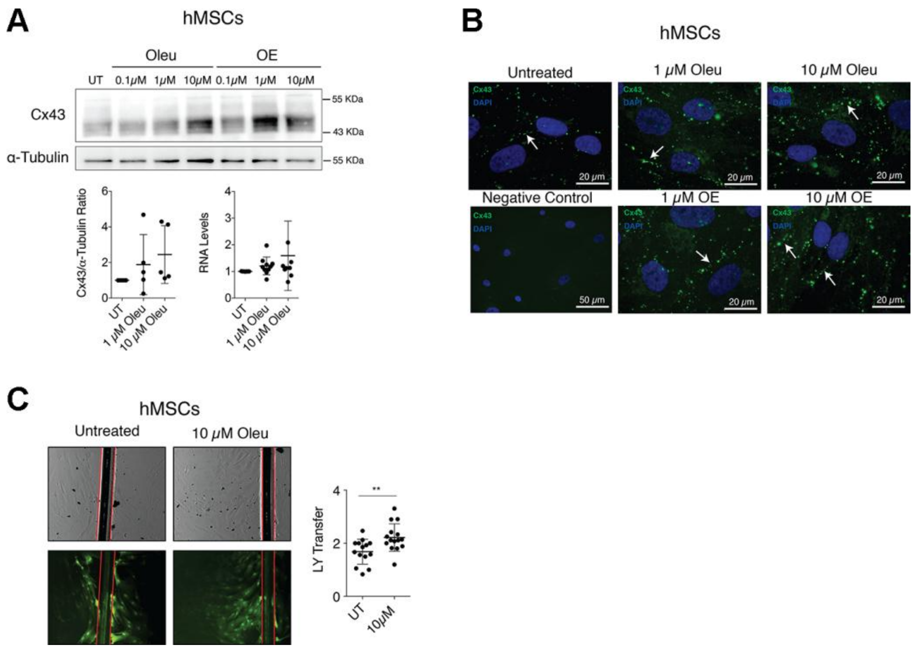


**Supplementary Figure 4.** (**A**) Western blot showing increased Cx43 protein expression in hMSCs obtained from bone marrow and subcutaneous inguinal fat and treated for 2 h with oleuropein or olive-extract (OE) containing 41.5% Oleu as well as other polyphenolic compounds. α-tubulin was used as a loading control (n=5 independent experiments; one-way ANOVA, *P*=0.2723). RT-qPCR showing increased Cx43 mRNA in hMSCs treated with Oleu in basal/growth medium (α-MEM with 10% FBS) (n=9 independent experiments; data were normalized to HPRT-1 levels; one-way ANOVA, *P*=0.1041). (**B**) Immunofluorescence analysis showing increased Cx43 at the membrane (GJ plaques, white arrows) in hMSCs treated with oleuropein or OE for 2 h. Cell nuclei were stained with DAPI. Original magnifications ×40 and ×100. Images represent two independent experiments. (**C**) SL/DT assay examining GJ activity in hMSCs treated with 10 µM Oleu or 10 µM OE. Red lines represent the cut edge where cells took up the LY immediately after scraping. The graph represents the ratio of stained cells to the number of cells at the scrape edge (n=10 independent experiments; Student's *t* test, *P*=0.0087). Data is expressed as mean±SD, **P*<0.05, ***P*<0.01 and ****P*<0.0001.

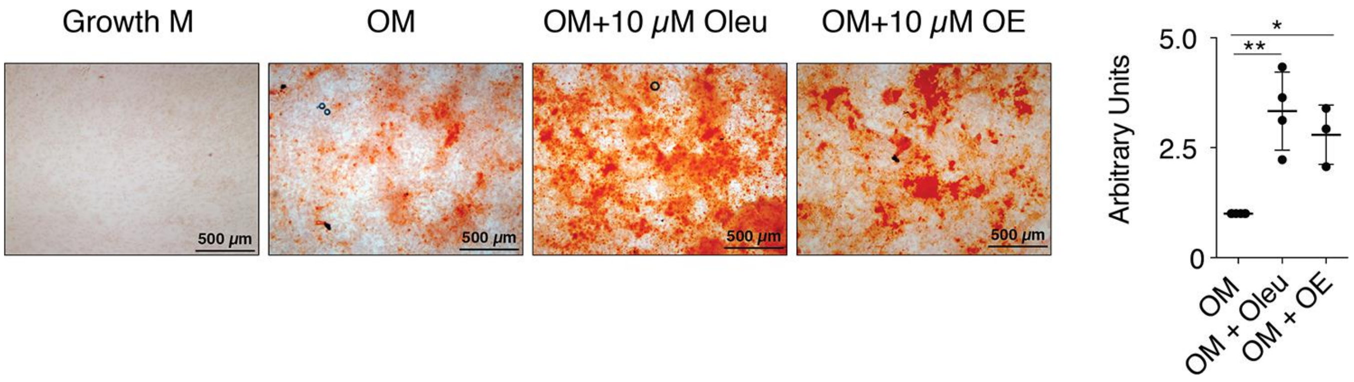


**Supplementary Figure 5. Calcium deposits in osteogenic medium-differentiated OACs evaluated by alizarin red staining.** OACs were cultured in osteogenic medium (OM) supplemented with 10 µM oleuropein (Oleu) or OE for 21 days. Quantification was performed by counting red pixels and normalization to hMSCs differentiated in osteogenic medium without treatment. n=3 technical replicates; mean±SD; one-way ANOVA with *P*=0.0023.

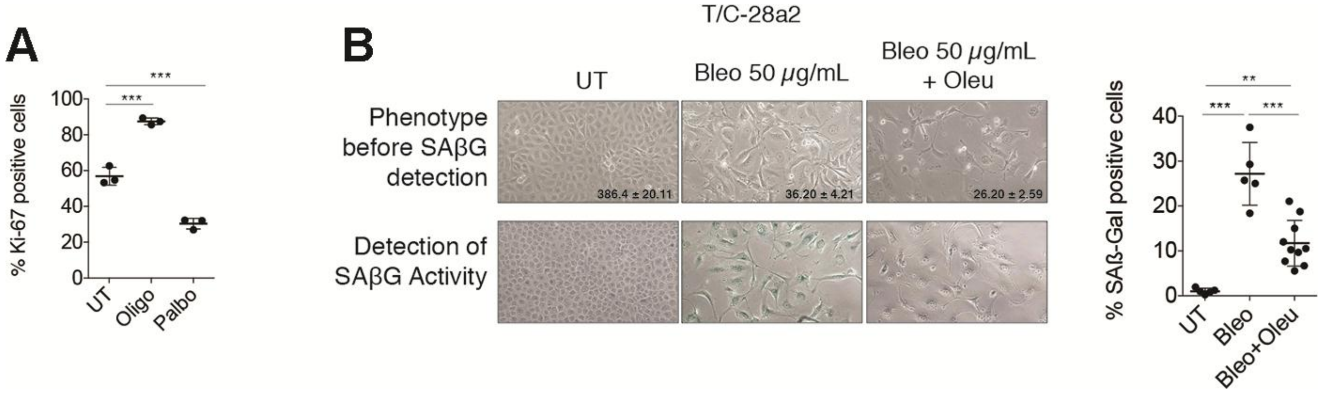


**Supplementary Figure 6.** (**A**) Cell proliferation was evaluated in the T/C-28a2 chondrocyte cell line by Ki-67 immunofluorescence after oligomycin or Palbociclib treatment (n=3 independent experiments; mean±SD; one-way ANOVA with *P*<0.0001). (**B**) T/C-28a2 chondrocyte cell line was cultured with 50 µg/mL of bleomycin for 24 h, and then cultured in normal growth medium or in the presence of oleuropein 10 µg/mL for another 24 h. The mean mean±SD of the number of cells from 5 different visual fields are shown. ß-galactosidase activity was detected by X-Gal cleavage, and cell staining (blue) was evaluated by microscopy. Quantification is shown on the right (n=5-10 technical replicates; mean±SD; one-way ANOVA with *P*<0.0001).
