# Supplementary material for: Senolytic activity of small molecular polyphenols from olive restores chondrocyte redifferentiation and promotes a pro-regenerative environment in osteoarthritis
Source: Aging (Albany NY). 2020 Aug 3;12(16):15882–905. doi: 10.18632/aging.103801 (PMC7485729; doi:10.18632/aging.103801)
Supplement: Supplementary Tables [file aging-12-103801-s001..pdf]

SUPPLEMENTARY TABLES

Supplementary Table 1. List of antibodies used in flow cytometry assays.

Antibody	Fluorochrome	Reference	Source	Laser (nm)	Filter	Dilution
CD105	PE	105PE-100T	Immunostep	488	585/42	1:50
CD166	APC	1399990314	Immunostep	635	661/16	1:100
Cx43	APC	FAB7737A	R&D	635	661/16	1:50

Supplementary Table 2. List of primer sequences (5'–3') used for RT-PCR analysis.

Gene name (protein name)	Forward	Reverse
ACAN (Aggrecan)	CAGAACAACTCGGGGAACAT	GCACAATTGGAACCCTGACT
Cdkn2a ($p16^{Ink4a}$)	GAGCAGAACGATAGGGCTTG	CATGTGCCCTCTCCTCCTAA
GJA1 (Cx43)	ACATGGGTGACTGGAGCGCC	ATGATCTGCAGGACCCAGAA
HPRT-1 (HPRT-1)	TTGAGTTTGGAAACATCTGGAG	GCCCAAAGGGAACTGATAGTC
IL-1ß (IL-1ß)	CGAATCTCCGACCACCACTAC	TCCATGGCCACAACAACTGA
IL-6 (IL-6)	TGTAGCCGCCCCACACA	GGATGTACCGAATTTGTTTGTA
MMP-3 (MMP-3)	CCCTGGGTCTCTTTCACTCA	GCTGACAGCATCAAAGGACA
CDH2 (N-cadherin)	TATTTCCATCCTGCGTGTGA	GCGTTTCATCCATACCACAA
OSTCN (Osteocalcin)	CCATGAGAGCCCTCACACTCC	GGTCAGCCAACTCGTCACAGTC
PPARG (PPARγ)	GCGATTCCTTCACTGATACACTG	GAGTGGGAGTGGTCTTCCATTAC
PTGS2 (COX-2)	CTTCACGCATCAGTTTTTCAAG	TCACCGTAAATATGATTTAAGTCCAC
TWIST1 (Twist-1)	CATGTCCGCGTCCCACTA	CACGCCCTGTTTCTTTGAAT
VIM (Vimentin)	ACTTTGCCGTTGAAGCTG	AATCCAGATTAGTTTCCCTCAGGT
